# p16^ink4a^ Positivity of Melanocytes in Non-Segmental Vitiligo

**DOI:** 10.3390/diagnostics10110878

**Published:** 2020-10-28

**Authors:** Jin Wook Lee, Tae Hyung Kim, Tae Jun Park, Hee Young Kang

**Affiliations:** 1 Department of Medical Sciences, Ajou University Graduate School of Medicine, Suwon 443–721, Korea; jinwooklee276@gmail.com; 2 Department of Dermatology, College of Medicine, Chungbuk National University, Cheongju 28644, Korea; 3 Department of Dermatology, Ajou University School of Medicine, Suwon 443–721, Korea; derma-tang@naver.com; 4 Department of Biochemistry and Molecular Biology, Ajou University School of Medicine, Suwon 443–721, Korea; 5 Institute on Ageing, Ajou University Medical Center, Suwon 443–721, Korea

**Keywords:** melanocyte, p16^INK4A^, senescence, vitiligo

## Abstract

Cellular senescence is induced in response to cellular stressors such as increased levels of reactive oxygen species. The chronic accumulation of senescent cells is currently recognized as a contributor to the pathologic processes of diverse degenerative diseases. Vitiligo is characterized by the disappearance of melanocytes driven by cellular stress within melanocytes and autoimmune processes. In this study, we examined p16^INK4A^ positivity in the lesional and perilesional skin of 54 non-segmental vitiligo patients to explore cellular senescence in vitiligo. There were more p16^INK4A^-positive melanocytes in the perilesional vitiligo skin samples than in control samples. It was also found that p16^INK4A^ immunoreactivity was not restricted to melanocytes but also existed in fibroblasts; the number of p16^INK4A^-positive fibroblasts was significantly increased in lesional skin compared to perilesional skin and normal controls. However, in the subgroup analysis of sun-exposed and non-exposed samples, this outcome was only found at sun-exposed sites, suggesting that fibroblast senescence is an epiphenomenon related to the loss of pigment in skin with vitiligo. In summary, exploring p16^INK4A^ positivity in vitiligo revealed melanocyte senescence in perilesional skin, which may play a role in vitiligo pathogenesis.

## 1. Introduction

Cellular senescence can be induced in response to cellular stressors such as UV irradiation and increased levels of reactive oxygen species (ROS), or as a result of telomere shortening. It is characterized by an irreversible cell-cycle arrest, which is accompanied by a number of phenotypic changes, such as the development of a senescence-associated secretory phenotype (SASP) [1]. Although senescent cells are normally cleared by the immune system, the chronic accumulation of such cells has been causally implicated in the pathologic processes of several degenerative diseases. [2,3,4]. The detrimental effects of senescent cells, such as chronic inflammation and disruptions of tissue structures, can be largely attributed to the SASP, which releases a number of pro-inflammatory cytokines, chemokines and growth factors.

Vitiligo is an acquired hypopigmentary disorder characterized by the progressive disappearance of melanocytes [5]. An autoimmune mechanism mediated by autoreactive CD8+ T cells that engage melanocytes is considered to be responsible for melanocyte loss in vitiligo. Research into the pathogenesis of vitiligo points to the importance of ROS and intrinsic melanocyte abnormalities as possible key inducers of the inflammatory cascade [6,7]. ROS can act as danger signals and activate pattern recognition receptors to initiate inflammation [8].

Notably, the long-term effects of subcytotoxic oxidative stress overlap with biomarkers of the pre-senescent cellular phenotype. Several studies have suggested that there are cellular and functional alterations of different cell populations in both lesional and perilesional areas of vitiligo skin. Deregulated intracellular redox homeostasis promotes the acquisition of a stress-induced premature senescent-like phenotype in melanocytes or keratinocytes cultured from perilesional vitiligo skin [9,10,11,12]. The few studies that exist on fibroblast abnormalities in the dermis of vitiligo patients show that perilesional vitiligo fibroblasts display increased basal ROS levels associated with the upregulation of the stress-induced marker p53 [12]. The relative gene expression levels of senescence markers are increased in the lesional dermis of non-segmental vitiligo (NSV) patients as compared with controls [13]. However, these results are mostly obtained from cultured cells, and there are only a few in vivo studies.

Therefore, consistent with the idea that the acquisition of stress-related modifications could be extended to the entire skin, we aimed to investigate senescence marker expression in the lesional and perilesional skin tissue of non-segmental vitiligo patients. We focused on the analysis of the senescence marker, endogenous CDK inhibitor p16^INK4A^, because staining for p16^INK4A^ has been considered one of the best in vivo and in vitro markers of cellular senescence [14] and a senescence effector protein in both fibroblasts and melanocytes of the skin [15,16]. Another CDK inhibitor, p21, might not be appropriate as its expression is downregulated in senescent melanocytes, which also indicates a lack of activation of p53 in the ‘replicative’ senescent melanocytes [15].

## 2. Materials and Methods

### 2.1. Patients

Fifty four patients with newly diagnosed, clinicopathologically-confirmed NSV were enrolled. Patients with associated autoimmune disorders or other inflammatory skin disorders were excluded from the study. Lesional and perilesional punch biopsies were collected from each patient. Perilesional skin was taken more than 5 cm away from the lesion margin. Normal skin samples were collected from the skin of patients receiving dermatologic surgery as healthy controls. This study was approved by the institutional review board of Ajou University Hospital (MED-MDB-20-215), approved on 3 July 2020.

### 2.2. Stains and Immunohistochemistry

Formalin-fixed paraffin-embedded tissue sections were stained with hematoxylin and eosin for light microscopic evaluation. Melanin pigment was visualized with a Fontana–Masson stain performed in the usual fashion. The epidermal and dermal cells were stained by immunohistochemistry using standard technique. We used the following antibodies: NKI/beteb (1:20, Monosan, Uden, The Netherlands), MART-1 (1:100, Neomarker, Fremont, CA, USA) to visualize melanocytes, and anti-p16^INK4A^ rabbit monoclonal antibodies (1:100, 725–4713, Ventana Medical Systems, Inc., Oro Valley, AZ, USA) to visualize senescent cells.

For tissue immunofluorescence, serial sections (4 μm) derived from formalin-fixed and paraffin-embedded blocks were dewaxed in xylene and rehydrated through graded ethanol to PBS. Tissue sections were incubated with the following primary antibodies: anti-p16^INK4A^ rabbit monoclonal antibodies (1:100, 725–4713, Ventana Medical Systems, Inc., Tucson, AZ, USA) and anti-MITF mouse monoclonal antibodies (1:100, MS-772-PO, Thermo Fisher Scientific, Fremont, CA, USA).

### 2.3. Imaging Analysis

The densities of p16^INK4A^-positive cells in the dermis were evaluated by light microscopic counting of stained cells in combination with measurement of dermal area by an interactive image-analysis system (Image Pro Plus, Version 4.5; Media Cybertics Co., Silver Spring, MD, USA). Cells showing p16^INK4A^ positivity were counted in skin sections and were normalized per mm^2^. The number of p16^INK4A^-positive cells in the measured dermal area was defined as cells within 300 μm from the dermal-epidermal junction. The number of p16^INK4A^-positive melanocytes was estimated by measuring the number of p16^INK4A^-positive melanocytes per 100 melanocytes on the basement membrane. First, the total number of melanocytes in the area was counted and normalized per mm. Melanocytes showing p16^INK4A^ positivity were also counted and normalized per mm. Next, the number of the p16^INK4A^-positive melanocytes was divided by the total number of the melanocytes per unit length, and then was multiplied by 100.

### 2.4. Statistical Analysis

Data are expressed as the mean ± standard error and were analyzed using Student’s *t*-test, paired Student’s *t*-tests, Mann–Whitney test, or Wilcoxon signed-rank test with a *p* value < 0.05 considered significant. No adjustments were made for multiple comparisons in the subgroup analysis. IBM SPSS ver. 23 (IBM Corp., Armonk, NY, USA) was used for all statistical analysis.

## 3. Results

### 3.1. Clinical Findings

This study included 54 patients with NSV, 25 (46.3%) males and 29 (53.7%) females, for a M:F ratio of 1:1.2. The patients’ ages at presentation ranged from 2 to 69 years (mean 30.2 years, median 26.0 years). The control group, with normal skin, consisted of 33 patients, 15 (45.5%) males and 18 (54.5%) females, for a M:F ratio of 1:1.2. The age of the control group ranged from 1 to 66 years (mean 30.9 years, median 32.0 years). The skin biopsy samples were divided into “young” (under age 20) and “adult” groups and were also classified into two subgroups, denoted as the sun-exposed and non-exposed groups. Among the 54 cases of NSV, 43 (79.6%) skin samples were collected from sun-exposed sites. The other 11 (20.4%) were from non-exposed sites. Twenty-seven samples (81.8%) of the 33 control cases were obtained from sun-exposed sites, and six (18.2%) were from non-exposed sites (Table 1).

### 3.2. Perilesional Melanocytes of NSV Displayed the Expression of the p16 ^INK4A^ Senescence Marker

**Figure 1:**
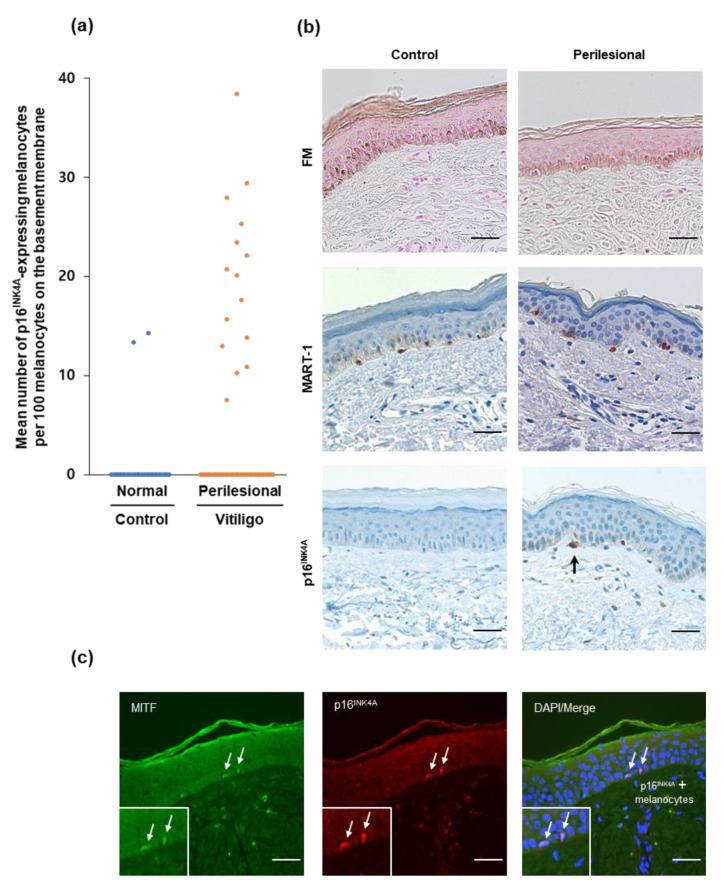
Number of p16^INK4A^-positive melanocytes in the perilesional epidermis of NSV patients and in normal control skin samples: (**a**) P16^INK4A^-positive melanocytes were quantified per 100 melanocytes on the basement membrane of a skin specimen. Data are expressed the mean ± SE, *n* = 54 patients with NSV, *n* = 33 normal control donors; *p* = 0.0023 according to a Student’s *t*-test. Raw data are available in Tables S1 and S2. (**b**) The amount of melanin was similar between NSV patients and normal controls. The melanocyte marker, MART1, showed no significant difference in melanocytic density count. A p16^INK4A^-positive melanocyte (arrow) is shown in the perilesional epidermis of NSV patients (scale bar, 100 μm). (**c**) Representative immunofluorescence reactivity of the p16^INK4A^ antibody in NSV. MITF (green)/p16^INK4A^ (red) double-immunostained melanocytes (arrows) are visualized.

To characterize cellular senescence in vitiligo skin, biopsies from lesional and perilesional normal skin of 54 vitiligo patients were assayed for a marker of cellular senescence, p16 ^INK4A^. There were more p16^INK4A^-positive melanocytes in the perilesional vitiligo skin than in the control samples (*p* = 0.0023 in a Student’s *t*-test) (Figure 1a). Among the 54 NSV patients, 15 cases showed p16^INK4A^-positive melanocytes (mean 5.5/100 melanocytes) in the perilesional epidermis. In the control skin samples, p16^INK4A^-positive melanocytes (mean 0.8/100 melanocytes) were observed in only two cases out of 33. Fontana–Masson (FM) staining showed similar amounts of melanin in both NSV patients and normal controls (Figure 1b). There was no significant difference in the overall number of melanocytes in the epidermis between the perilesional skin of NSV patients and the control samples (Figure S1). A p16^INK4A^/MITF double-immunostaining assessment revealed that the p16^INK4A^-positive senescent cells found were melanocytes (Figure 1c).

In a subgroup analysis of sun-exposed and non-exposed skin samples, p16^INK4A^-positive melanocytes were mostly observed in the sun-exposed group. There were more p16^INK4A^-positive melanocytes in the perilesional vitiligo skin (mean 6.9/100 melanocytes) than in the normal healthy skin samples (mean 1.0/100 melanocytes) (*p* = 0.008 according to a Mann–Whitney test). In an analysis of the young group, two of 22 young vitiligo cases (the youngest at age nine) showed p16^INK4A^-positive melanocytes in the perilesional epidermis. None of the melanocytes in the epidermis of the young control patients were positive for p16^INK4A^.

### 3.3. The Number of p16 ^INK4A^-Positive Fibroblasts Was Increased in the Exposed Skin of NSV Patients

**Figure 2:**
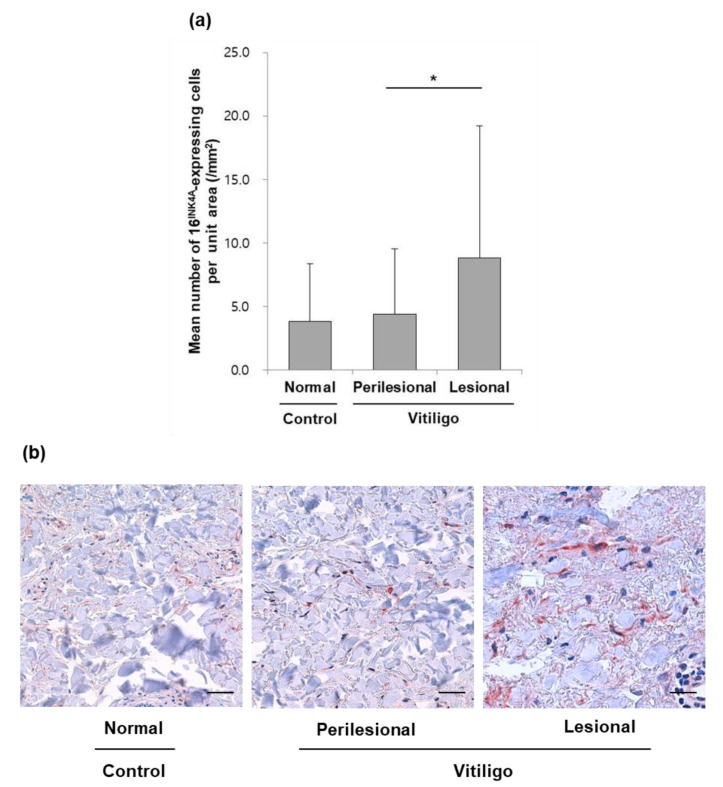
Number of p16^INK4A^-positive cells in the dermis: (**a**) p16^INK4A^ positive cells were quantified according to the measured dermal area (/mm^2^) of each skin specimen. These results are shown in the form of a comparison between all NSV patients and the normal controls. Data are presented as the mean ± SE, *n* = 54 patients with NSV, *n* = 33 normal controls; * *p* = 0.0035 from paired Student’s *t*-tests. (**b**) Senescence marker, p16^INK4A^, and expression levels from vitiligo tissue biopsies. The number of p16^INK4A^ positive cells was more significantly increased in the dermis of the lesional skin from NSV patients compared to perilesional skin or normal control skin samples (scale bar, 100 μm).

The number of p16^INK4A^-positive cells was significantly increased in the dermis of lesional skin from patients with NSV as compared to perilesional skin (*p* = 0.00035 from paired Student’s *t*-tests) (Figure 2). P16^INK4A^-positive cells were presumed to be fibroblasts based on the shape and distribution of the cells. There was no significant difference in p16^INK4A^ positivity in the dermis between perilesional NSV skin and normal control skin (Figure 2).

**Figure 3:**
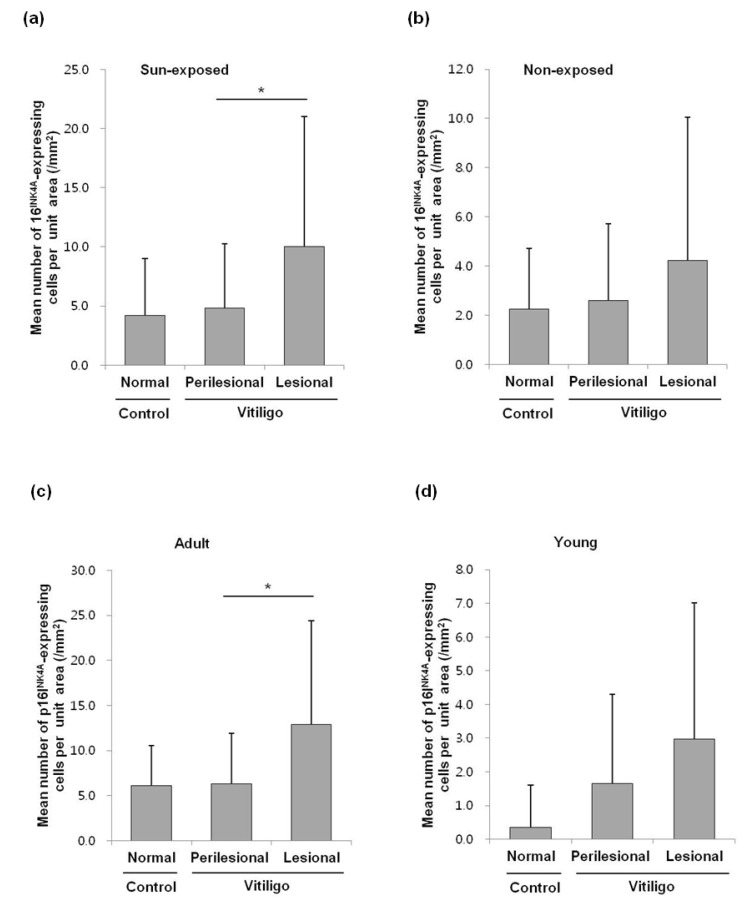
Subgroup analysis of the sun-exposed/non-exposed and the adult/young groups, quantified according to the measured dermal area (/mm^2^) of each skin specimen: (**a**) results from the sun-exposed subgroups; data are shown as the mean ± SE, *n* = 43 patients with NSV, *n* = 27 normal controls; * *p* = 0.0007 from paired Student’s *t*-tests. (**b**) Results from the non-exposed subgroups; data are shown as the mean ± SE, *n* = 11 patients with NSV, *n* = 6 normal controls; *p* > 0.05 from a Mann–Whitney test and a Wilcoxon signed-rank test. (**c**) Results from the adult subgroups; data are shown as the mean ± SE, *n* = 32 patients with NSV, *n* = 20 normal controls; * *p* = 0.0006 from paired Student’s *t*-tests. (**d**) Results from the young subgroups; data are shown as the mean ± SE, *n* = 22 patients with NSV, *n* = 13 normal controls; *p* > 0.05 from a Mann–Whitney test and a Wilcoxon signed-rank test.

In a subgroup analysis, the sun-exposed and the adult subgroups had the same statistically significant difference in the number of p16^INK4A^-positive cells as observed in the comparison between all NSV patients and the normal controls (Figure 3a,c). However, the non-exposed and the young subgroups had no statistically significant difference in the number of p16^INK4A^-positive cells (Figure 3b,d).

## 4. Discussion

Senescent melanocytes have been shown to accumulate in human skin with age. Recent reports have found that the number of melanocytes expressing p16^INK4A^ is higher in aged human skin (mean age 63.4) [17,18,19]. In our study, however, p16^INK4A^-positive melanocytes were observed in the young vitiligo skin samples (the youngest at age nine). There were also more p16^INK4A^-positive melanocytes in the perilesional vitiligo skin samples than in control samples. These results are consistent with a previous study in which vitiligo cells showed a significant increase in p16^INK4A^ uncorrelated with the chronological age of the vitiligo cell donor [9]. The study argues for intrinsic metabolic defects in perilesional vitiligo melanocytes, leading to intracellular oxidative stress, as the primary intracellular signal for melanocyte degeneration [9]. Accordingly, oxidative-stress-induced senescence can be proposed as a mechanism leading to increased cellular senescence in vitiligo [20]. Although ROS levels have not been examined in our study, the mitochondria have been posited as a possible site of increased ROS production in vitiligo, and an altered expression of complex I has been demonstrated in both the peripheral blood mononuclear cells of vitiligo patients and in melanocytes from perilesional skin [21,22].

Senescent cells can be recognized and eliminated by the immune system [23,24]. Therefore, the question arises as to why senescent melanocytes are not eliminated by the immune system. What role do they play in the development of vitiligo? Answers remain incomplete. Different immune cell types have been implicated in studies of senescent cells depending on the pathophysiological context [25,26,27]. Recent evidence has shown that SASP is partially responsible for the persistent low-level chronic inflammation known as sterile inflammation [28,29,30]. The high level of matrix metalloproteinase 3 produced by melanocytes has been suggested to explain the presence of senescent cells in non-lesional vitiligo skin [9]. SASP-induced tissue degeneration may help senescent cells to avoid immune clearance [31]. Moreover, SASP-induced chronic inflammation may favor normal melanocyte loss, possibly contributing to the development of vitiligo. The functional roles of senescent melanocytes in vitiligo pathogenesis require further study.

Interestingly, in our study, p16^INK4A^ positivity was not restricted to melanocytes but was also associated with keratinocytes (data not shown). Moreover, two out of the 22 young vitiligo cases here (under age 20) showed p16^INK4A^-positive keratinocytes in the perilesional epidermis (data not shown). None of the keratinocytes and melanocytes in the epidermis samples of the young normal controls showed a positive p16^INK4A^ result. A central role of keratinocytes in vitiligo has recently emerged, as it is well documented that they are damaged and that these cells represent the predominant source of chemokines, such as CXCL9 and CXCL10, involved in the recruitment of T cells to vitiligo skin [32]. The role of senescent keratinocytes in vitiligo pathogenesis also requires further investigations.

Fibroblasts are the most abundant dermal cells and the most commonly studied cell type in terms of cellular senescence. Premature senescent fibroblasts feature as an important element in the pathophysiology of hyperpigmentary disorders such as senile lentigo and melasma [13,33]. The secretory phenotype of senescent fibroblasts is thought to be responsible for inducing melanocyte activation, resulting in skin hyperpigmentation [33]. Paradoxically, in vitiligo, fibroblasts showing senescence markers have also been reported, which suggests that senescent fibroblasts play a pathogenic role in the hypopigmentation of vitiligo. In one study, fibroblasts expressing senescence markers (p16^INK4A^, p21 and hp1) were increased in the lesional dermis of NSV patients (*n* = 8, leg skin) compared to perilesional and healthy control skin samples [13]. Non-lesional vitiligo fibroblasts exhibiting a myofibroblast and a premature senescence phenotype have also been found in active NSV patients (*n* = 8, gluteal skin) [12].

In our study, we also found that the number of p16^INK4A^-positive fibroblasts was more significantly increased in lesional skin from NSV patients (*n* = 54) compared to perilesional skin and normal controls. However, when we divided each group into two subgroups, in this case sun-exposed and non-exposed groups, based on the anatomic sites where biopsies were taken, there was no significant difference in terms of p16^INK4A^ positivity in the non-exposed subgroups. In addition to chronological aging, it is well known that extrinsic factors such as chronic sun exposure can induce fibroblast senescence [16]. In vitro, UV-exposed fibroblasts express senescence biomarkers [16]. It is therefore speculated that the fibroblast alteration in vitiligo may stem from chronic UV accumulation accompanying the loss of pigment in the epidermis. Whether or not senescent fibroblasts play a pathogenic role in vitiligo with regard to SASP remains a subject for future study. Indeed, it has been suggested that the increased expression level of dickkopf1 (DKK1) in lesional fibroblasts is associated with a senescent melanocyte phenotype that favors melanocyte loss [34].

In summary, exploring p16^INK4A^ positivity in vitiligo revealed melanocyte senescence in perilesional skin, possibly playing a role in the pathogenesis of vitiligo.

## Figures and Tables

**Table 1:** Characteristics of NSV patients and normal controls.

Characteristic	NSV Patients		Normal Controls	
Sex, *n* (%)				
Male	25	(46.3)	15	(45.5)
Female	29	(53.7)	18	(54.5)
Age, *n* (years)				
Adult (≥20)	32	(43.9)	20	(46.8)
Young (<20)	22	(10.2)	13	(6.5)
Biopsy site, *n* (%)				
Sun-exposed	43	(79.6)	27	(81.8)
Non-exposed	11	(20.4)	6	(18.2)

## Supplementary Materials

The following are available online at https://www.mdpi.com/2075-4418/10/11/878/s1, Figure S1: Number of total melanocytes on the basement membrane in the perilesional epidermis of NSV patients and in normal control skin samples, Table S1: The raw data of 33 patients with normal skin, Table S2: The raw data of 54 patients with NSV.

## Abbreviations

CXCL9	Chemokine (C-X-C motif) ligand 9
CXCL10	Chemokine (C-X-C motif) ligand 10
DKK1	Dickkopf1
MART-1	Melanoma antigen recognized by T cells 1
MITF	Microphthalmia-associated transcription factor
NKI/beteb	Anti-melanoma gp100 antibody
NSV	Non-segmental vitiligo
qRT-PCR	Quantitative real-time polymerase chain reaction
ROS	Reactive oxygen species
Smac/DIABLO	Second mitochondria-derived activator of caspase/direct inhibitor of apoptosis-binding protein with low pI
