# Supplementary material for: p16ink4a Positivity of Melanocytes in Non-Segmental Vitiligo
Source: Diagnostics (Basel). 2020 Oct 28;10(11):878. doi: 10.3390/diagnostics10110878 (PMC7694005; doi:10.3390/diagnostics10110878)
Supplement: Supplementary file 1 [file diagnostics-10-00878-s001.pdf]

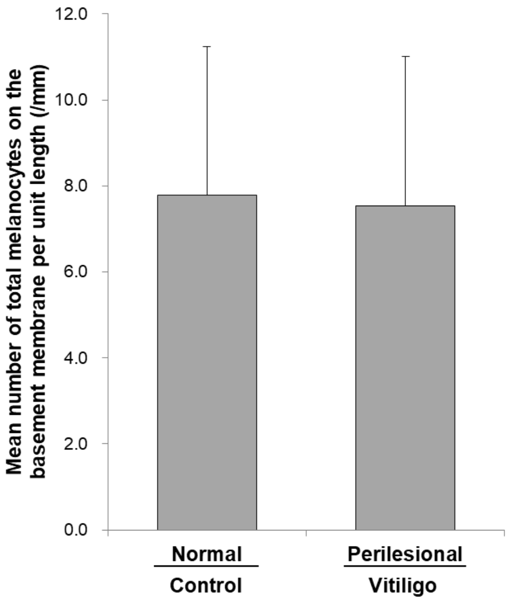

Figure 1. Number of total melanocytes on the basement membrane in the perilesional epidermis of NSV patients and in normal control skin samples. The number of total melanocytes was quantified per unit length (/mm). Data are expressed the mean ± SE, n = 54 patients with NSV, n = 33 normal control donors; p = 0.756 according to a Student's t-test.

Table S1. The raw data of 33 patients with normal skin.

	Sex	Age	Number of p16 + MCs (/mm)	Number of Total MCs (/mm)	Number of p16 + MCs (/100 MCs)	Number of p16 + FBs (/mm^2)
1	F	66	1.0	7.0	14.3	9.5
2	M	65	0.0	8.4	0.0	13.2
3	M	62	0.0	13.2	0.0	4.7
4	M	61	0.0	7.3	0.0	0.0
5	F	58	0.0	9.9	0.0	8.5
6	M	56	0.0	9.6	0.0	0.0
7	F	54	0.0	8.8	0.0	9.5
8	F	53	0.0	2.5	0.0	8.7
9	F	51	2.3	17.0	13.3	9.1
10	F	48	0.0	15.5	0.0	13.1
11	F	46	0.0	7.4	0.0	4.2
12	F	43	0.0	12.1	0.0	10.7
13	F	42	0.0	6.2	0.0	4.4
14	M	40	0.0	4.9	0.0	0.0
15	F	34	0.0	5.7	0.0	8.5
16	M	34	0.0	6.2	0.0	4.5
17	M	32	0.0	10.8	0.0	0.0
18	F	32	0.0	8.7	0.0	0.0
19	F	32	0.0	3.3	0.0	4.4
20	F	27	0.0	2.9	0.0	9.1
21	M	13	0.0	8.7	0.0	0.0
22	F	11	0.0	6.7	0.0	4.6
23	M	10	0.0	10.2	0.0	0.0
24	M	10	0.0	9.1	0.0	0.0
25	M	8	0.0	9.3	0.0	0.0
26	F	6	0.0	8.2	0.0	0.0
27	M	6	0.0	8.4	0.0	0.0

28	M	5	0.0	6.2	0.0	0.0
29	F	5	0.0	5.6	0.0	0.0
30	M	4	0.0	6.6	0.0	0.0
31	F	3	0.0	4.7	0.0	0.0
32	F	3	0.0	2.6	0.0	0.0
33	M	1	0.0	2.8	0.0	0.0
**Mean**		30.9	0.1	7.8	0.8	3.8
**SE**		22.3	0.4	3.5	3.3	4.5

P16^{INK4A}-positive melanocytes and the total melanocytes on the basement membrane were quantified per the measured basement membrane length (/mm) of a skin specimen. P16^{INK4A}-positive melanocytes were also quantified per 100 melanocytes on the basement membrane (/100 MCs) of a skin specimen. P16^{INK4A}-positive fibroblasts in the dermis were quantified according to the measured dermal area (/mm^2) of each skin specimen. MCs: melanocytes; FBs: fibroblasts.

Table S2. The raw data of 54 patients with NSV.

			Perilesion				Lesion	
	**Sex**	**Age**	**Number of p16 + MCs (/mm)**	**Number of Total MCs (/mm)**	**Number of p16 + MCs (/100 MCs)**	**Number of p16 + FBs (/mm^2)**	**Number of p16 + MCs (/mm)**	**Number of p16 + FBs (/mm^2)**
1	F	69	0.0	2.6	0.0	4.7	0.0	4.3
2	F	68	0.0	11.7	0.0	8.5	0.0	8.3
3	M	67	2.3	13.0	17.6	13.1	0.0	49.9
4	F	63	2.5	8.4	29.4	8.6	0.0	12.8
5	M	62	0.0	2.5	0.0	4.4	0.0	7.9
6	F	61	0.0	3.4	0.0	13.1	0.0	26.3
7	M	58	2.7	10.6	25.3	4.7	0.0	13.2
8	F	55	0.0	6.4	0.0	4.3	0.0	34.8
9	F	54	0.0	3.7	0.0	9.5	0.0	4.7
10	M	54	2.0	14.7	13.8	0.0	0.0	4.3
11	M	53	4.1	10.8	38.4	15.5	0.0	38.8
12	M	50	0.0	13.3	0.0	13.1	0.0	13.0
13	F	49	0.0	10.3	0.0	8.8	0.0	13.1
14	F	47	0.0	3.1	0.0	0.0	0.0	8.7
15	F	47	0.0	5.9	0.0	0.0	0.0	0.0
16	F	47	1.3	5.6	23.4	20.2	0.0	12.8
17	M	45	2.2	11.2	20.1	12.7	0.0	4.5
18	M	43	0.0	7.3	0.0	12.6	0.0	25.0
19	M	43	2.5	11.3	22.1	5.0	0.0	8.9
20	F	39	0.0	13.0	0.0	8.7	0.0	15.9
21	F	38	2.3	8.4	27.9	8.6	0.0	21.7
22	M	34	0.0	5.9	0.0	0.0	0.0	0.0
23	F	31	0.9	9.1	10.2	0.0	0.0	4.4
24	M	31	0.0	13.0	0.0	8.8	0.0	4.2
25	M	30	0.0	5.0	0.0	0.0	0.0	0.0
26	M	26	0.0	13.4	0.0	0.0	0.0	11.0
27	F	26	0.0	7.1	0.0	8.7	0.0	13.1
28	F	26	1.1	7.2	15.7	4.5	0.0	8.9
29	M	25	0.0	7.7	0.0	0.0	0.0	11.1
30	F	23	0.0	8.0	0.0	4.3	0.0	17.8
31	F	21	1.0	4.7	20.7	0.0	0.0	4.4
32	F	20	1.1	10.4	10.9	0.0	0.0	8.8
33	F	19	0.0	6.8	0.0	0.0	0.0	0.0
34	M	17	0.0	3.4	0.0	0.0	0.0	14.7
35	F	17	0.0	5.8	0.0	9.3	0.0	0.0
36	F	16	0.0	9.0	0.0	4.3	0.0	0.0
37	M	16	0.0	11.8	0.0	4.6	0.0	4.5
38	M	14	1.0	13.4	7.5	0.0	0.0	0.0

39	F	13	0.0	4.2	0.0	4.4	0.0	5.3
40	F	13	0.0	3.0	0.0	0.0	0.0	0.0
41	F	12	0.0	8.9	0.0	0.0	0.0	4.4
42	M	12	0.0	3.5	0.0	0.0	0.0	9.4
43	F	10	0.0	6.8	0.0	0.0	0.0	0.0
44	M	9	0.0	4.6	0.0	4.4	0.0	4.8
45	M	9	0.0	7.8	0.0	0.0	0.0	9.2
46	F	9	0.9	7.0	12.9	4.8	0.0	4.5
47	M	8	0.0	4.3	0.0	0.0	0.0	0.0
48	M	6	0.0	3.6	0.0	0.0	0.0	0.0
49	F	6	0.0	8.2	0.0	0.0	0.0	0.0
50	F	6	0.0	4.1	0.0	0.0	0.0	4.3
51	M	5	0.0	4.6	0.0	0.0	0.0	0.0
52	M	3	0.0	9.8	0.0	4.4	0.0	0.0
53	M	3	0.0	2.8	0.0	0.0	0.0	4.4
54	F	2	0.0	4.9	0.0	0.0	0.0	0.0
**Mean**		30.2	0.5	7.5	5.5	4.4	0.0	8.9
**SE**		20.5	1.0	3.5	9.9	5.1	0.0	10.4

$P16^{INK4A}$-positive melanocytes and the total melanocytes on the basement membrane were quantified per the measured basement membrane length (/mm) of a skin specimen. $P16^{INK4A}$-positive melanocytes were also quantified per 100 melanocytes on the basement membrane (/100 MCs) of a skin specimen. $P16^{INK4A}$-positive fibroblasts in the dermis were quantified according to the measured dermal area (/mm^2) of each skin specimen. MCs: melanocytes; FBs: fibroblasts.
